# Prevention and Reduction of Anxiety in Autistic Preschoolers Through an Autism-Specific Parent-Mediated Intervention: A Pilot Randomised Controlled Trial Evaluating Short and Longer Term Outcomes

**DOI:** 10.1007/s10803-024-06570-5

**Published:** 2024-09-26

**Authors:** Dawn Adams, Stephanie Malone, Nicole Dargue, Deb Keen, Jacqui Rodgers, Kate Simpson, Rachelle Wicks, Ashleigh Bullot, Ron Rapee

**Affiliations:** 1 Autism Centre of Excellence, Griffith University, Messines Ridge Road, Mt Gravatt, Brisbane, QLD 4122 Australia; 2 Griffith Institute for Educational Research, Brisbane, QLD Australia; 3 Population Health Sciences Institute, Newcastle University, Newcastle Upon Tyne, UK; 4 Centre for Emotional Health, Department of Psychology, Macquarie University, Sydney, Australia

**Keywords:** Autism, Mental health, Caregiver, Young children

## Abstract

Anxiety is a common co-occurring condition for autistic preschoolers. Whilst there has been extensive research evaluating anxiety prevention/reduction interventions for neurotypical preschoolers, such research is limited for autistic children. Fifty-seven parents of autistic 4–5-year olds, with varying levels of anxiety, participated in a randomised controlled trial of an autism-specific, parent-mediated intervention (CLK-CUES) to prevent or reduce anxiety in autistic preschoolers. Baseline, short-term (post-intervention) and longer term (12 months follow-up) assessments included child anxiety (ASC-ASD-P and PAS-R), intolerance of uncertainty, and parent well-being. (Trial registration ACTRN12620001322921). There were no reports of harmful effects of the intervention or trial. Linear mixed models show a significant group x time interaction for ASC-ASD-P Total score and the Uncertainty subscale with medium and large effect sizes. Post-hoc analyses show a significant decline for only the intervention group in anxiety (specifically, anxiety around uncertainty) from pre-post intervention, maintained at one year follow-up. There was no change on the PAS-R or other ASC-ASD-P subscales. CLK-CUES shows promise as a way to prevent and reduce anxiety in young autistic children, specifically anxiety related to uncertainty. Trials with larger samples are warranted. Findings also highlight the importance of using measures designed for autistic children.

**Supplementary Information:**

The online version contains supplementary material available at 10.1007/s10803-024-06570-5.

The most frequently reported mental health diagnosis amongst autistic people is anxiety, with meta-analyses suggesting that approximately 40% of autistic youth will have a clinical diagnosis of an anxiety disorder (van Steensel et al., 2011). However, community-based self-report and parent-report studies have suggested that a larger proportion of autistic youth, between 53% and 83%, experience anxiety at a level that significantly impacts their daily life (Adams et al., 2019b, 2020). Community-based samples using both qualitative and quantitative methodologies have highlighted that elevated anxiety levels (not specifically clinically diagnosed anxiety) can have a significant impact on the lives of autistic people and of those who support them. These increased levels are associated with poorer quality of life (Adams, Clark Adams et al., 2019a, b; Lin & Huang, 2019), higher levels of anger and behavioural challenges (Kerns et al., 2015; Townsend et al., 2022), and lower levels of participation in general (Ambrose et al., 2021), even in enjoyable or preferred activities (Ong et al., 2023). The combination of this elevated prevalence and significant impact on a wide range of life outcomes highlights the critical need to identify effective interventions to reduce anxiety in autistic people.

## Anxiety in Autistic Preschoolers

Research to date has provided evidence of elevated anxiety in autistic preschoolers (64%) compared to neurotypical preschoolers (44%; Klein et al., 2023). These prevalence rates of anxiety in autistic preschoolers are similar to those reported in school-aged autistic children (Chan et al., 2021). Longitudinal studies of anxiety in young autistic children suggest that the most common trajectory of anxiety symptoms is that once they are present, they increase over time (Baribeau et al., 2021). These studies also suggest that autism-related characteristics, such as insistence on sameness and repetitive behaviours, may predate or predict later anxiety symptoms (Baribeau et al., 2020, 2022). The differing presentation and autism-related predictors of anxiety in younger autistic children, as well as its potential to increase in symptom severity over time, indicate a need for interventions aimed at reducing anxiety in its earliest stages and preventing anxiety onset in the future.

There is an extensive body of literature describing skills-based interventions that aim to prevent and/or reduce anxiety in neurotypical children as young as 2 years old, with a meta-analysis reporting significant moderate effects of such interventions to reduce anxiety symptoms (Ooi et al., 2022). Some of these programs have been trialled in young autistic children. The Being Brave program, loosely modelled on the Coping Cat program, was evaluated in an open pilot study resulting in a significant reduction in anxiety in 16 autistic children aged 3–7 years with at least one anxiety disorder (mean 5.7 years; Driscoll et al., 2020). However, as this intervention uses cognitive behavioural therapy (CBT) approaches, the child participants had to have an IQ of 80 or above and phrase speech or above. Another program designed for neurotypical children that has been piloted in parents of young autistic children is Cool Little Kids (CLK). CLK is a six-session, parent-mediated program that has demonstrated excellent outcomes in the reduction and prevention of anxiety among preschool-aged neurotypical children, with benefits of the intervention being noted through to adolescence (Rapee, 2013). Bischof et al. (2018) showed that CLK also led to significant reductions in anxiety among a group of 26 preschool-aged autistic children, with feedback from parents suggesting that future offerings of the intervention could use more autism-specific examples and information. Given that the focus of CLK is on young children and on preventing anxiety, parents can participate in CLK even if their child does not have a diagnosis of an anxiety disorder. Such interventions could potentially address the need for mental supports that are accessible for autistic young people and their family members before mental health challenges reach the level at which a clinical diagnosis is made (Mandy, 2022).

## The Case for Mental Health Supports Designed for Autistic Young People

The need for mental health supports that are relevant to autistic people has been identified as a critical research priority (Brede et al., 2022). Meta-analyses (e.g., Sharma et al., 2021; Ung et al., 2015) have shown a moderate effect size for some CBT-based anxiety interventions for school-aged autistic youth (e.g., Facing Your Fears, Coping Cat, Cool Kids). However, CBT-based anxiety interventions specifically adapted to include autism-related aspects and experiences of mental health problems are more effective than non-adapted approaches (Wood et al., 2020). In their review of research relating to anxiety in young autistic children (aged 6 years or below), Vasa et al. (2020) identified only four intervention studies, three of which used CBT-based, parent-mediated approaches; however, only one of these CBT-based approaches was specifically adapted for young autistic preschoolers and none had alternative treatment controls. Vasa et al. concluded that there is a need to develop specific anxiety interventions for autistic children in this preschool age group which specifically target potential risk factors for anxiety in autistic children.

One well-documented risk factor for anxiety in autistic children that can be incorporated into interventions for anxiety in autistic preschoolers is intolerance of uncertainty (IU). IU is a transdiagnostic cognitive vulnerability that has been shown to explain significant variance in anxiety experienced by autistic people (Jenkinson et al., 2020). Although not unique to autism, IU has been identified as an important interrelated construct in the development and maintenance of anxiety in preschool-aged autistic children. A meta-analysis of 16 studies (*n* = 1,353 participants) found a large positive correlation of *r* = .62 for the relationship between IU and anxiety, with moderator analyses concluding that this relationship is strongest in young autistic children, a relationship not reported in non-autistic children (Bird et al., 2024). Vasa et al. (2023) identified IU as the only direct correlate of child anxiety in a network analysis approach in 75 autistic preschoolers, with other correlates such as sensory over-responsivity being modulated by IU. However, the relationship between anxiety and other child variables is likely to be complex and multifaceted, so further work with larger samples is needed to further explore potential correlates and their interrelationships. Whilst IU is experienced by the child, it impacts upon parenting approaches, with parents of autistic preschoolers describing how they avoid uncertain situations and aim to create predictability and certainty due to their child’s difficulties with IU (Ong et al., 2023).

Not only is IU strongly linked to anxiety in autistic preschoolers, it has also been linked to intervention outcomes. Keefer et al. (2016) found that youth with higher levels of baseline IU responded less positively to modified CBT. This suggests that interventions for anxiety in autistic children may be enhanced by targeting IU within treatment. Rodgers et al. (2019) has developed a parent-mediated intervention (Coping with Uncertainty in Everyday Situations; CUES) for anxiety in autistic children which specifically addresses the risk factor of IU. CUES showed positive effects in a feasibility study, indicating promise for the effectiveness of parent-mediated, autism-specific interventions for reducing anxiety in autistic children, as well as potentially reducing anxiety and other internalising problems in the parents of the autistic children (Rodgers et al., 2023). Further work is now needed to develop and trial interventions which address anxiety through addressing the risk factor of IU for preschool-aged autistic children.

## This Study

With the increased recognition of elevated anxiety in autistic preschoolers (e.g., Keen et al., 2019) and the finding that, once present, anxiety symptoms tend to increase over time (Baribeau et al., 2021), there is a clear need to develop effective interventions to reduce or prevent anxiety symptomatology in autistic preschoolers. To address unmet needs identified by the autistic and autism communities, such interventions need to consider the underlying constructs shown to be related to anxiety in autistic people (i.e., IU). As noted above, CUES was specifically developed to reduce IU in autistic children. CUES is an eight-session, parent-mediated intervention which focuses on helping parents of autistic children (aged 6–16 years) to support their child to be able to tolerate and cope with everyday uncertain situations that make them anxious. CUES includes psychoeducation to help parents recognise IU, enables parents to identify potential developmental and environmental factors that may trigger IU for their child, and teaches parents to plan and use appropriate strategies aimed at increasing their child’s tolerance of uncertainty. Each parent identifies a chosen target IU situation to focus on across the weeks and identifies strategies from the CUES materials to help improve outcomes in that situation. The initial trial of CUES reports a reduction in child anxiety and improvements in the target IU situation for the intervention over the control group (Rodgers et al., 2023). However, to date, the standard CUES has not been evaluated in autistic preschoolers. As discussed above, CLK has strong evidence for reducing and preventing anxiety in neurotypical preschoolers but is not tailored to autistic children. Therefore, for this study, we combined CLK and CUES to create an intervention tailored to prevent and reduce anxiety in young autistic people: CLK-CUES.

The aims of this trial were therefore (a) to evaluate whether CLK-CUES, a parent-mediated, autism-specific intervention to prevent or reduce anxiety in preschool autistic children with varying levels of anxiety, is effective at reducing child anxiety, child IU, and/or parental internalising problems at short or longer-term follow-up; and (b) to determine whether there were any negative or harmful effects of the CLK-CUES.

Based on the evidence available to date, we hypothesised that children whose parents received CLK-CUES (intervention group) would have significantly lower scores at short-term (5 months) and longer term (≈ 15 months) follow-ups than those in the “usual care” (control group) on measures of (a) child anxiety, (b) child IU, and (c) parent internalising problems.

## Methods

This paper reports on a randomised control trial evaluating the efficacy of the autism-specific anxiety intervention. It was registered with Australian New Zealand Clinical Trials Registry (ANZCTR; number ACTRN12620001322921). Human Research Ethics Committee approval was granted by Griffith University HREC committee (reference 2019/989). There is a published protocol: Adams et al. (2021).

### Study Design

Participants were recruited into a randomised controlled trial. The intervention group received CLK-CUES and the control condition received no intervention from the study team (i.e., treatment as usual). Participants attended the research centre to complete face-to-face and questionnaire assessments at Time 1 (T1)/baseline, at Time 2 (T2)/short-term follow-up/post-intervention (x̅ = 4.98 months post-baseline, *SD* = 2.08 months), and at Time 3 (T3)/longer term follow-up (x̅ = 14.9 months post-baseline, *SD* = 3.42 months). T1 and T2 were conducted before the child entered school, with T2 being scheduled 3–5 weeks after the intervention was completed. T3 was conducted after the child had transitioned into the first year of formal schooling. Participants were provided with gift vouchers after each timepoint to thank them for their time.

**Fig. 1:**
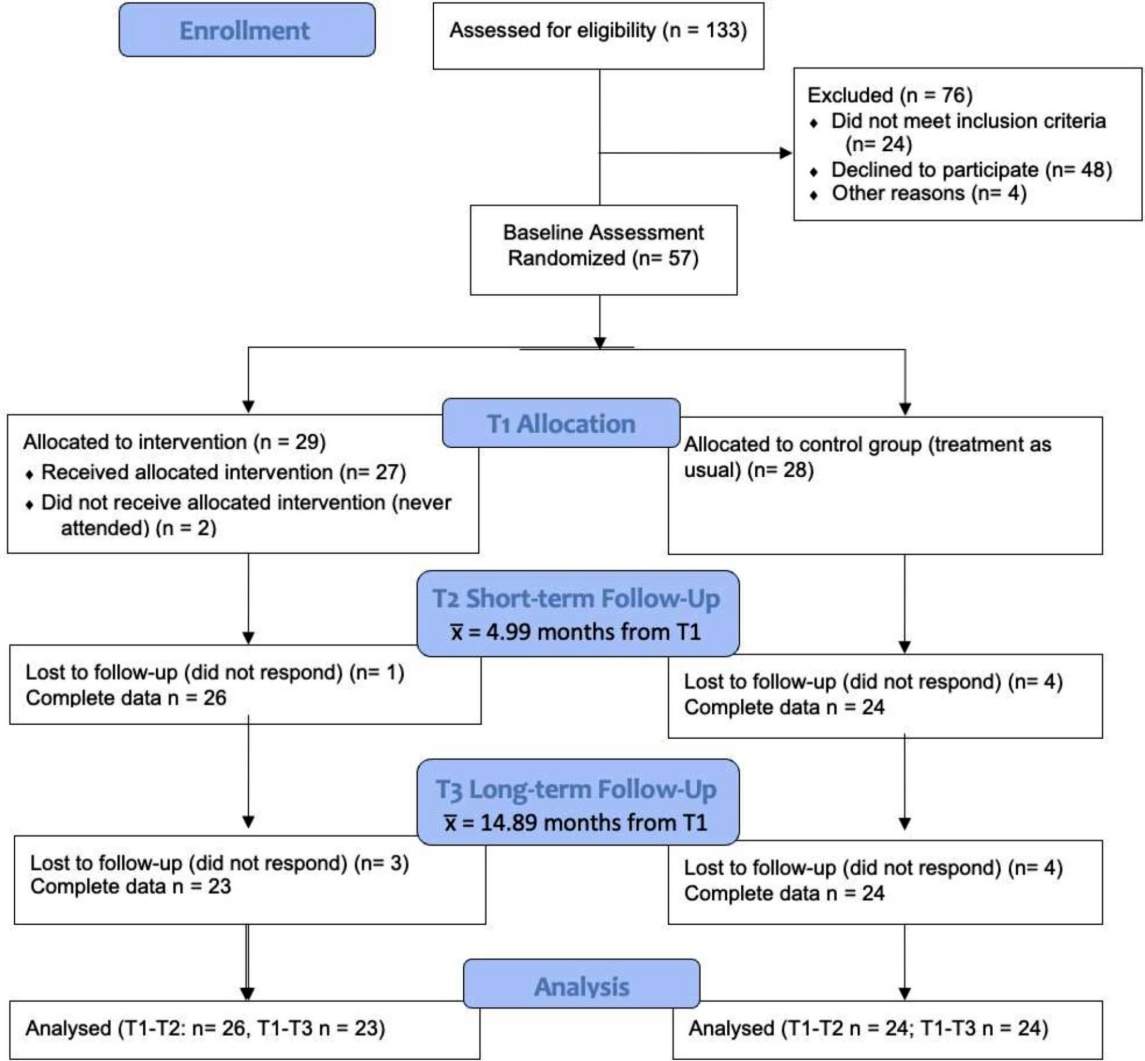
CONSORT diagram for study

Figure 1 summarises the aspects of the trial protocol that are reported upon in this paper. It visually depicts the features at each stage that were common to both the intervention and control groups, as well as the aspects that were unique to the intervention group. All participants who had completed T1 assessments were invited to attend T2 and T3 assessments.

### Trial Team

The trial team was set up to ensure those conducting the assessments remained uninformed to group allocation across the study. The team consisted of a project manager, two researchers who conducted the child assessments, and a clinician-researcher who delivered the intervention. The project manager was the only team member who was in contact with all participants and was aware of their group allocation. They arranged appointments and provided parents with questionnaires to complete but did not conduct any of the assessments with the children. The two researchers who conducted child assessments were not aware of the group allocation of participants. The clinician-researcher who delivered the intervention was not involved in any of the child assessments or the parent questionnaire data collection.

### Recruitment

Recruitment information was distributed through multiple methods to support inclusion of a broad range of participants, including social media, local television and radio shows, autism-specific and general early learning centres, parent and caregiver support groups, autism-specific schools, local mental health centres, and autism-specific psychology centres. Inclusion criteria stated that children must (1) be 4–5 years at the time of assessment, (2) have a diagnosis of autism (this was verified through the Autism Diagnostic Observation Schedule-2 [ADOS-2] at T1), and (3) not yet have commenced their first year of formal schooling. Parents also had to be able to attend face-to-face parent group sessions. There were no set exclusion criteria (i.e., children were not required to have a predetermined or clinical level of anxiety and were not excluded based on co-occurring diagnoses e.g., intellectual disability [ID]). An a priori power calculation (detailed in the trial registration) identified the need for 64 participants (32 in each group) to allow for a 90% retention rate and sufficient power to identify a medium effect size at a significance level of 5%.

Recruitment was conducted in waves across February 2020 to July 2021 to allow parent groups to occur during school term times. Recruitment began prior to the COVID-19 pandemic was declared; it was paused as soon as the lockdown restrictions were implemented and resumed once restrictions enabled these activities to happen. Parent groups began once public health measures allowed these to occur face-to-face. Once the parent groups began, there were three further short (3–7 days) lockdowns in the geographical area of the study, none of which occurred across the dates of the parent groups. Due to challenges in recruiting during the pandemic, the sample (described below) was slightly below the 64 participants required by the a priori power analysis; therefore, this could be considered a pilot study.

### Procedure

If parents expressed an interest in participating in the study, they were screened for eligibility and sent the information sheet and consent form. Of the 59 who provided informed consent to participate, 57 attended for an initial assessment at the research centre of the lead author. Once initial assessments for each recruitment wave were complete, the project manager used a random number generator to randomly allocate half of the families to the intervention condition (CLK-CUES parent-mediated anxiety intervention) and half to the control condition. The project manager notified parents of their group status via email and parents in the intervention condition were asked to select the workshop location and time most appropriate for them. Options included morning, afternoon, and evening groups across multiple locations to support parental attendance. Both parents were invited to attend the intervention sessions but only one parent attended for each participant. The parent who attended each session was encouraged to share the information, materials, and homework tasks with the coparent and/or other caregivers for the child.

### Participants

Table 1 shows the demographics of the children in the intervention and control groups. Table 2 shows the demographics of the caregiving parent who attended the groups.

**Table 1:** Child characteristics at baseline based on parent report; Mean (SD), or n (%)

		Control N = 28	CLK-CUES N = 29	Group comparison
Age (months) Mean (SD)	Baseline (T1)	58.75 (5.36)	57.00 (5.40)	*t*(55) = 1.21, *p* = .23, *d =* 0.32
	Short-term follow up (T2)	63.83 (4.99)	61.92 (6.00)	*t*(48) = 1,22, *p* = .23, *d =* 0.35
	Longer term follow-up (T3)	73.83 (4.76)	72.04 (6.00)	*t*(45) = 1.14, *p* = .26, *d =* 0.33
Sex	Male	20 (71.4%)	18 (62.1%)	*χ*^2^(1) = 0.56, *p* = .58^+^
	Female	8 (28.6%)	11 (37.9%)	
Ethnicity	Australian	17 (58.6%)	16 (57.1%)	*χ*^2^(5) = 4.69, *p* = .46
	First Nations/ Indigenous Australian	3 (10.7%)	1 (3.4%)	
	White/European	3 (10.7%)	6 (20.7%)	
	Asian	4 (14.3%)	2 (6.9%)	
	African	1 (3.6%)	0 (0%)	
	Not reported	1 (3.6%)	3 (10.3%)	
Co-occurring diagnoses at T1 (based on parent report)^1^				
Speech and language disorder	Formally diagnosed	9 (33.3%)	10 (35.7%)	*χ*^2(^2) = 2.12, *p* = .90
	Informally diagnosed	4 (14.8%)	3 (10.7%)	
	Not diagnosed	14 (51.9%)	15 (53.6%)	
Anxiety	Formally diagnosed	2 (8.0%)	7 (25.9%)	*χ*^2^(2) = 2.92, *p* = .23
	Informally diagnosed	14 (56.0%)	12 (44.4%)	
	Not diagnosed	9 (36.0%)	8 (29.6%)	
Intellectual disability	Formally diagnosed	1 (3.8%)	1 (3.7%)	*χ*^2^(2) = 0.01 *p* = 1.0
	Informally diagnosed	1 (3.8%)	1 (3.7%)	
	Not diagnosed	24 (92.3%)	25 (92.6%)	
Attention Deficit Hyperactivity Disorder (ADHD/ADD)	Formally diagnosed	3 (11.5%)	5 (18.5%)	*χ*^2^(2) = 0.1.01, *p* = .60
	Informally diagnosed	5 (19.2%)	3 (11.1%)	
	Not diagnosed	18 (69.2%)	19 (70.4%)	

*Note* ^1^Some parents chose not to answer so percentages are calculated from those who answered

**Table 2:** Demographics of the parent who attended the groups, as reported at baseline^1^

		Control N = 28	CLK-CUES N = 29	Group comparison
Group attendee	Mother	25 (89.3%)	22 (78.6%)	*χ*^2^(2) = 1.69, *p* = .43
	Father	3 (10.7%)	5 (17.9%)	
	Caregiver	0 (0%)	1 (3.6%)	
Age at T1	21–30 years	5 (17.9%)	1 (3.6%)	*χ*^2^(4) = 5.17, *p* = .27
	31–40 years	14 (50%)	18 (64.3%)	
	41–50 years	8 (28.6%)	8 (28.6%)	
	51–60 years	0 (0%)	1 (3.6%)	
	61 or above	1 (3.6%)	0 (0%)	
Ethnicity	Australian	13 (50%)	13 (48.1%)	*χ*^2^(6) = 5.27, *p* = .51
	First Nations/ Indigenous Australian	2 (7.7%)	0 (0%)	
	Other oceanic	1 (3.8%)	1 (3.7%)	
	White/European	5 (19.2%)	9 (33.3%)	
	Asian	4 (15.4%)	3 (11.1%)	
	African	1 (3.8%)	0 (0%)	
	South American	0 (0%)	1 (3.7%)	
Family income (pre-tax, AUD$)	< $37,000	6 (21.4%)	2 (6.9%)	*U* = 310, *p* = .22
	$37,001–-$80,000	4 (14.3%)	3 (10.3%)	
	$80,001–$180,000	12 (42.9%)	15 (51.7%)	
	> $180,001	6 (21.4%)	7 (24.1%)	
Highest level of education	Secondary school	7 (75%)	21 (25%)	*χ*^2^(1) = 1.02, *p* = .31
	Tertiary education	4 (14.3%)	24 (85.7%)	
Disclosed diagnoses	Autism	5 (17.9%)	2 (7.1%)	*χ*^2^(1) = 1.47, *p* = .23
	Speech and language disorder	1 (3.6%)	1 (3.6%)	*χ*^2^(1) = 0.00, *p* = 1.00
	Mental health disorder	8 (28.6%)	12 (42.9%)	*χ*^2^(1) = 1.24, *p* = .27
	Neurodevelopmental condition (intellectual disability, dyspraxia, ADHD/ADD)	2 (7.1%)	2 (7.1%)	*χ*^2^(1) = 0.00, *p* = 1.00

*Note* ^1^Some parents chose not to answer some questions so percentages are calculated from those who answered

### Measures

In addition to demographic questionnaires, all parents completed the following outcome measures at all timepoints.

#### Bespoke Questionnaires

Parents completed purpose-designed questions about services or supports accessed for their child’s anxiety. As monitoring of adverse or harmful events related to the intervention was a secondary outcome, parents in the intervention group were asked to report on any negative events or adverse reactions to the activities experienced during or after each session. As adverse events can take weeks or months to show, parents were also asked to report on any negative outcomes when returning for the T2 and T3 assessments.

#### Primary Outcome: Child Anxiety

Two parent-report measures of anxiety were used to measure outcomes. The *Anxiety Scale for Children - Autism Spectrum Disorder Parent report* (ASC-ASD-P; Rodgers et al., 2016) is a 24-item, autism-specific measure of anxiety which was originally designed for ages 8 and over but has been used in samples as young as 5 years of age (Keen et al., 2019). The ASC-ASD-P consists of four subscales: Performance anxiety (5 items), Anxious arousal (6 items), Separation anxiety (5 items), and Uncertainty (8 items), with a higher score representing higher levels of anxiety. This is an anxiety scale rather than a diagnostic assessment. No clinical cut-offs have been suggested or validated for this age group. The ASC-ASD-P has good to excellent validity with other measures (den Houting et al., 2019), and good 1-month test–retest reliability (*r* = .84, ICC = 0.84; Rodgers et al., 2016). Cronbach’s alpha across the three timepoints in this study ranged from 0.89 to 0.91 for Performance anxiety, 0.65 to 0.71 for Anxious arousal, 0.70 to 0.78 for Separation, 0.86 to 0.87 for Uncertainty, and 0.88 to 0.92 for the Total score. As the Anxious arousal subscale score was below acceptable levels of reliability (< 0.7) it was not used in analyses.

A second measure of anxiety, the Preschool Anxiety Scale Revised (PAS-R; Edwards et al., 2010), was also administered. Although not autism specific, the PAS-R has previously been used to measure anxiety in intervention trials with young autistic children (Bischoff et al., 2018). The subscales included were Generalised anxiety (5 items), Social anxiety (6 items), and Separation anxiety (5 items), alongside the Total score (28 items). The PAS-R is not a diagnostic instrument but can be used to evaluate change over time in response to treatment or prevention programs. Across the three timepoints, Cronbach’s alpha for this sample ranged from 0.67 to 0.72 for the Generalised anxiety subscale, 0.71 to 0.82 for the Social anxiety subscale, 0.67 to 0.75 for the Separation anxiety subscale, and 0.90 to 0.95 for the Total score. As the Generalised anxiety and Separation anxiety subscale scores were both below acceptable levels of reliability (< 0.7), these were not included in the analyses.

#### Secondary Outcome: Intolerance of Uncertainty

To measure cognitive and emotional reactions to uncertainty, two measures of IU were included. The Responses to Uncertainty and Low Environmental Structure (RULES; Sanchez et al., 2017) is a 17-item, parent-report measure developed to measure the need for rules and certainty for children aged 3–10 years. Whilst age relevant to this sample, it has not been widely used or validated in autistic samples. For this reason, the 12-item Intolerance of Uncertainty Scale – Parent version (IUS-P; Walker, 2009) was also included. This was adapted by Boulter et al. (2014) and has been frequently used to describe IU in school-aged autistic children (e.g., Neil et al., 2016). Cronbach’s alpha was α = 0.93 at every time point for the RULES and α = 0.95 for the IUS-P.

#### Secondary Outcome: Parent Internalising Problems

To evaluate whether the CLK-CUES intervention impacted upon parental well-being, parents completed the Depression Anxiety Stress Scales (DASS-21; Lovibond & Lovibond, 1995). This is widely used in clinical and non-clinical samples, including for parents of autistic children. The DASS distinguishes between three separate factors: Depression, Anxiety (fear, panic), and Stress (tension, agitation). Cut-off points are set for mild/moderate/severe/extreme for each scale. Cronbach’s alpha at each timepoint ranged from 0.86 to 0.91 for the Depression subscale, 0.78 to 0.88 for the Anxiety subscale, and 0.83 to 0.91 for the Stress subscale.

### Intervention

The development of the intervention is described in detail in the published protocol (Adams et al., 2021). Briefly, the program was an amalgamation of two parent interventions: CLK, which was amended for autistic children, with added content from CUES. For example, the initial session of standard CLK includes information on anxiety, types of anxiety, information on what leads to anxiety in non-autistic preschoolers, and the role of parent anxiety in child anxiety. CLK-CUES covers all of these topics and also included information on autism itself, information on how some aspects of the autism profile (e.g., sensory processing differences) can lead to anxiety, and how anxiety may present differently in young autistic children. Additionally, in the later sessions, content from CUES is added in to enable parents to reflect on the potential role of IU in their child’s anxiety. Parents are set additional homework tasks to build confidence and tolerance of IU in a way that is safe and respectful for a young autistic person.

The resulting CLK-CUES intervention consisted of six sessions, each 2 h long, covering specific predefined topics. Parents received a workbook for each session and a folder for storing these documents so they could be referred to at any time. Each session began by asking parents to report back on their homework tasks and to reflect on successes and barriers as a group. Parents attended face-to-face unless they were unwell, in which case they were able to videoconference into the session or arrange a catch-up session with the clinician-researcher. The workbook summarised the information covered, detailed the out-of-session activities to be completed, and provided worksheets for parents to complete before and after activities. The CLK-CUES intervention was piloted with a small group of parents with a qualitative and visual inspection of quantitative data suggesting positive impact (Simpson et al., 2023). The intervention was refined based on feedback from the parents in the pilot group prior to use in this trial.

Families in the control arm received care as usual. This was selected as the comparator to indicate what would have been the common outcome had the intervention not been implemented. No restrictions were placed on what any child or parent could or could not access outside of the study. As described in the measures, parents were asked to report on the supports accessed between assessments so that this could be described for the control and intervention groups.

Fidelity of the intervention group was checked through therapist-completion of a session topic checklist. Additionally, an independent rater reviewed 20% of recorded group intervention sessions to rate for fidelity to the treatment manual, using the session topic checklist developed for the study.

### Data Analyses

Measures were scored as per the manuals, with pro-rating and imputation of missing data as recommended. Data were screened for normality. Where skew and kurtosis values were not within acceptable limits, data were transformed and rescreened for skew and kurtosis, with all statistics being +/- 2. Due to the exploratory nature of the study, alpha was set at 0.05.

To address the first aim of the study and to evaluate whether CLK-CUES results in short-term and/or longer-term reductions in child anxiety and parental internalising difficulties, a series of linear mixed models (LMMs) were constructed using intention to treat (ITT) analysis. Walker et al. (2019) provided an informative description of LMMs and their use in clinical research. For each model, the outcome measure was the score on the child anxiety, IU, or parent internalising problems questionnaire. Fixed factors were group (intervention or control) and timepoint (T1/2/3), with participant included as a random factor. The model was fitted using maximum likelihood.

LMMs are an extension of the linear regression model and include random effects and correlated errors. LMMs allow for the examination of fixed effects while partitioning the variance (i.e., error) that is due to individual differences, enabling greater statistical control within participants compared to traditional methods. Further, variance due to individual variation can be controlled within the modelling process by including participant as a random effect. LMMs have the advantage over repeated measures ANOVA in that they do not exclude participants if one datapoint is missing because the observed values are used to determine the longitudinal trajectory through the use of random effects.

LMMs have been used in previous studies evaluating the standard CLK intervention (e.g., Doyle et al., 2021). As per these previous studies, where significant effects are noted, planned contrasts will compare change from T1 to the T2/T3 between conditions as well as T1, T2, and T3 scores between groups. Effect sizes of these planned comparisons are reported as Cohen’s *d*, with 0.2 representing a small effect size (“noticeably smaller than medium but not so small as to be trivial”), 0.5 a medium effect size (“representative of an effect likely to be visible to the naked eye of a careful observer”), and 0.8 a large effect size (“the same distance above the medium as small is below it”; Cohen, 1998, p. 25).

To address the second aim of the trial, which was to identify any harms or adverse outcomes from the intervention, the number of adverse events or negative reactions to the intervention or activities undertaken were reported for each session and across the duration of the intervention.

### Community Involvement

Autistic and autism community members were involved in all stages of the research. The study team (i.e., those who contributed to the study design, development, and/or analysis) and manuscript authors include autistic and neurodivergent adults, family members and friends of autistic individuals, and those working in support organisations for autistic people. The intervention was piloted with members of the autism community, with their feedback used to refine the materials.

## Results

### Parent Attendance and Completion of Measures

As shown in Fig. 1, of the 57 parents and children who completed T1 assessments, seven (three from the intervention group, four from the control group) did not complete T2 assessments. This included two participants who did not begin the intervention and did not respond to the research team’s attempts to contact them to identify their reason for non-attendance. All 57 parents were reinvited to return for T3 assessments (in line with the ITT analysis plan). From the 57 parents who completed T1, 10 did not return for T3: six from the intervention group (which includes the two parents who did not attend any intervention sessions) and four from the control group. Those with complete data (i.e., from T1, T2, and T3) did not differ from those with incomplete data (i.e., missing data from T2, T3, or both) on measures of child anxiety (ASC-ASD-P Total: *t*(55) – 1.16, *p* = .25; PAS Total *t*(30.18) = 1.31, *p* = .20), parent internalising problems (DASS Stress *t*(53) – 0.93, *p* = .36; Anxiety *t*(54) = 0.34, *p* = .47; Depression *t*(54) = 0.21, *p* = .37), or parent education (*X*^2^(1) = 0.02, *p* = .89). However, participants with one or more missing timepoints had significantly lower family income than those with complete data (*U* = 150, *p* = .04). Approximately one third of participants who attended all timepoints had household incomes below AUD$80,000 (pre-tax) per annum whereas over 80% of those who missed T2, T3, or both had incomes in this category.

All but one of the parents who began the intervention attended all six sessions (whether in person, teleconferenced in, or attended a teleconference catch up). That one parent only missed one session without a teleconference catch due to family circumstances, and this was the final session of the intervention. The final session does not present new material but provides a recap, allows parents to ask questions or discuss any unresolved queries, and asks parents to set goals or intentions for themselves and/or their child (linked to the intervention) into the future. Every parent reported back on homework completion each week.

### Child External Supports During Trial

Parents reported therapies and supports accessed at T1, T2, and T3. The therapists accessed were predominantly stable across the study. The number of children from the control group reported to be seeing a clinical psychologist was 11 (T1), 10 (T2), and 11 (T3). The number of children from the intervention group reported to be seeing a clinical psychologist was 10 (T1), 9 (T2), and 9 (T3), as one child (in the intervention group) stopped seeing their psychologist after the intervention. Two children moved from intensive therapy settings into school, so new allied health therapist(s) were engaged at that time. Seven children began taking new medications between T2 and T3 (which covers the time the children transitioned into school). Four of these were from the control group (3 risperidone, 1 fluoxetine) and three from the intervention group (2 fluoxetine, 1 sertraline).

### Effect of CLK Intervention on Outcome Measures

**Fig. 2:**
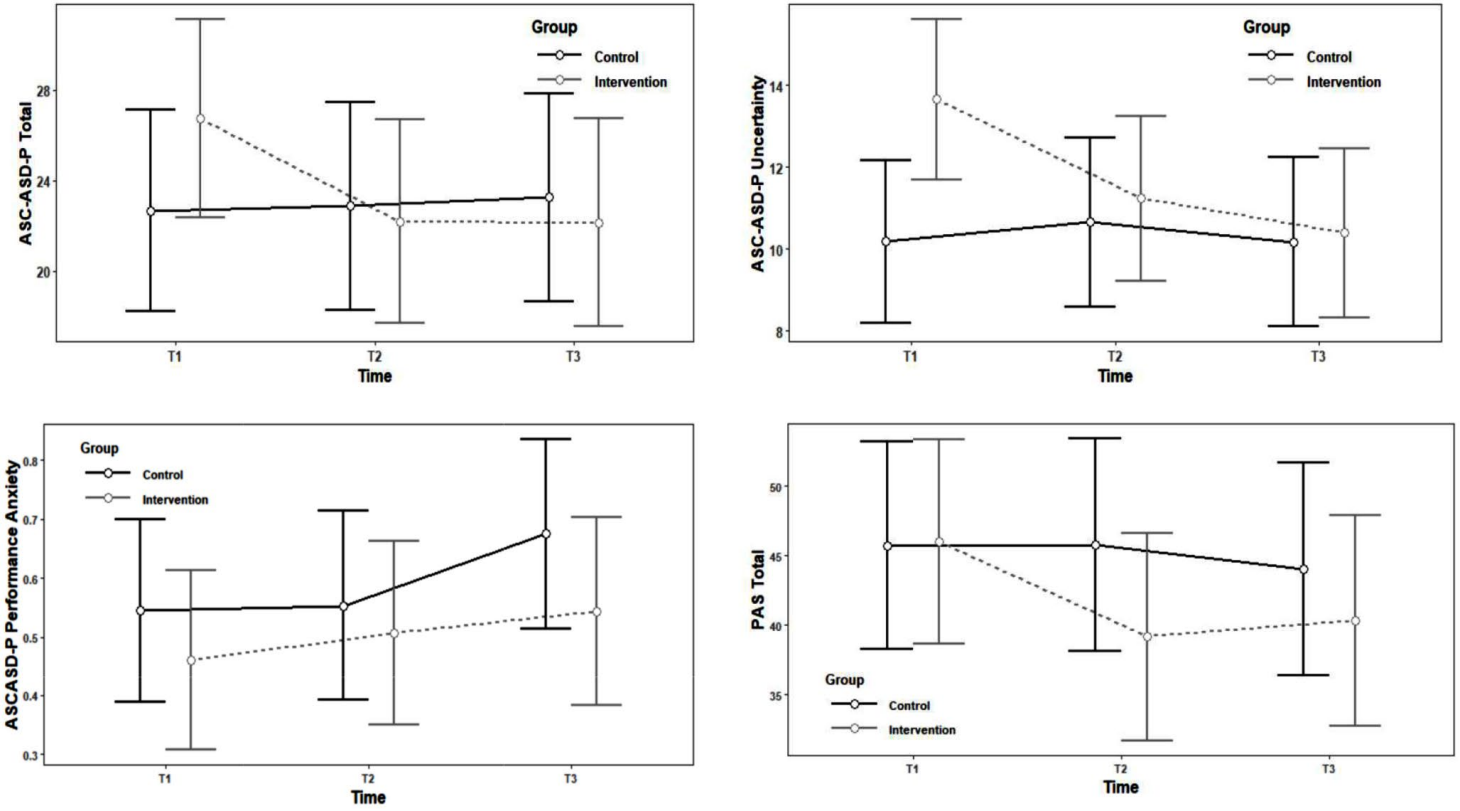
Outcome measures for control and intervention groups T1 (pre-intervention), T2 (short-term follow up post-intervention), and T3 (longer term follow up) (i) ASC-ASD-P Total Score (ii) ASC-ASD-P Uncertainty Subscale Score (iii) ASC-ASD-P Performance Anxiety Subscale and (iv) PAS Total Score. Note. Y axes have different scaling for ease of visual inspection

The results of the LMMs for the primary outcome measures are summarised in Table 3, alongside the mean score, standard deviations and between group effect sizes at each time point. These data for the secondary outcomes are in the Supplementary Table 1. Significant interactions were present in two of the eight scores within the primary outcome of child anxiety; the ASC-ASD-P Total and ASC-ASD-P Uncertainty subscale scores. Additionally, a significant main effect of Time was shown for the ASC-ASD-P Uncertainty and Performance Anxiety subscales, and the PAS-Total and Social Anxiety subscale scores. These are described further below and graphically represented in Fig. 2. All other models for secondary outcomes (child IU and parent internalising problems) were non-significant. For reference, correlations between outcome variables at each timepoint are presented in Supplementary Tables 2–4.

**Table 3:** Mean (*SD*) of primary outcomes for control and intervention group at each time point and size of between-group effects, with results of Linear mixed models

Variable		Time 1			Time 2			Time 3			Results of Linear Mixed Model (LMM)		
		Control *M(SD)*	Intervention *M(SD)*	Between-group effect size	Control *M(SD)*	Intervention *M(SD)*	Between-group effect size	Control *M(SD)*	Intervention *M(SD)*	Between-group effect Size	Time	Group	Group*Time
ASC-ASD-P	Total	22.68 (11.59)	26.76 (12.89)	− 0.68	23.08 (11.79)	22.81 (12.83)	0.12	23.37 (11.97)	23.52 (10.30)	0.19	F(2, 95.52) = 211.70, *p* = .13	F(1, 55.98) = 1.25, *p* = .27	F(2, 96.32) = 4.73, *p* = .01**
	Uncertainty	10.18 (4.97)	13.66 (5.38)	-1.19	10.83 (5.26)	11.50 (5.81)	− 0.20	10.33 (5.81)	10.83 (4.89)	− 0.07	F(2, 95.63) = 2.06, *p* = .13	F(1, 55.50) = 0.77, *p* = .47	F(2, 95.63) = 0.44, *p* = .65
	Performance^1^	4.07 (3.84)	3.69 (4.11)	0.39	3.71 (3.58)	3.73 (3.86)	0.22	5.17 (4.03)	4.26 (3.78)	0.60	F(2, 95.84) = 0.54, *p* = .58	F(1, 55,35) = 0.58, *p* = .45	F(2, 95.84) = 1.85, *p* = .16
	Separation	5.07 (3.61)	6.38 (3.19)	− 0.68	5.58 (3.72)	5.23 (3.41)	0.08	5.17 (2.93)	6.17 (3.59)	− 0.36	F(2, 96.32) = 3.90, *p* = .02*	F(1, 55.98) = 1.25, *p* = .27	F(2, 96.32) = 4.73, *p* = .01**
PAS-R	Total	45.71 (21.04)	45.97 (22.29)	− 0.03	46.29 (19.59)	39.85 (19.23)	0.82	43.21 (20.48)	42.61 (17.43)	0.46	F(2, 94.78) = 3.19, *p* = .046*	F(1, 55.59) = 0.45, *p* = .50	F(2, 94.78) = 2.30, *p* = .11
	Social Anxiety Subscale	10.75 (6.07)	11.41 (7.11)	− 0.23	10.33 (5.65)	9.96 (5.94)	0.17	9.88 (5.61)	9.78 (5.85)	0.06	F(2,95.27) = 3.51, *p* = .03*	F(1, 55.52) = 0.00, *p* = 1.00	F(2, 95.27) = 0.54, *p* = .58

^1^ Scores were transformed for analysis but raw scores presented here. Effect sizes calculated using transformed scores

### Primary Outcome: ASC-ASD-P Total

Overall, the model of Group, Time, and their interaction (with participant included as a random factor) accounted for a substantial amount of variance in ASC-ASD-P Total scores (conditional *R*^*2*^ = 0.75), with 2% of the variance explained by the fixed effects alone. Within this model, the Time x Group interaction was statistically significant, *F*(2, 96.32) = 4.73, *p* = .01). Planned within-group post-hoc comparisons indicated that the intervention group had a medium effect size reduction in anxiety from T1-T2 (*d* = 0.76) and T1-T3 (*d* = 0.77) whereas there was no significant change for the control from T1-T2 (*d* = 0.04) or T1-T3 (*d* = 0.01). As per Table 3, between-group comparisons highlight a medium between-group effect size at T1 (*M*_*diff*_ = -4.08; *d = −* 0.68), post-intervention (T1 and T2), the between-group effect size was negligible (*d =* 0.12; d = 0.19). Barnett et al.’s (2004) process of reducing the effect of regression to the mean on outcome variables was followed. This showed that the treatment effect on the ASC-ASD-P Total score, after controlling for regression to the mean, is -3.55 for T1-T2 and − 4.08 for T1-T3.

### ASC-ASD-P Uncertainty

Overall, the model of Group, Time, and their interaction (with participant included as a random factor) accounted for a substantial amount of variance in ASC-ASD-P Uncertainty scores (conditional *R*^*2*^ = 0.71), with 6% of the variance explained by the fixed effects alone (marginal *R*^*2*^). Within this model, the Group x Time interaction (*F*(2, 96.32) = 4.73, *p* = .01) and the main effect of Time (*F*(2, 96.33) = 3.90, *p* = .02) were statistically significant. Planned within-group post-hoc group comparisons showed a significant decrease in ASC-ASD-P Uncertainty scores for the intervention group from T1 to T3 with a large effect size, (t(96.5) = 3.87, *p* = .003, *d* = 1.11). In contrast, there was no significant change over time in scores on the Uncertainty scale for the control group, (t(95.5) = 0.004, *p* = 1.00, *d* = 0.001). As per Fig. 2; Table 3, the intervention group were significantly higher than the control group at baseline (*M*_*diff*_ = -3.48; *d =* -1.19) but post-intervention (T1 and T2), their anxiety had reduced such that it was not significantly different from the control group (*M*_*diff*_ = -0.21; *d =* -0.07). We used Barnett et al.’s (2004) process to evaluate treatment effect whilst statistically taking into account potential regression to the mean. The treatment effect on the ASC-ASD-P Uncertainty subscale score, after controlling for regression to the mean, is -1.51 for T1-T2 and − 3.13 for T1-T3.

### ASC-ASD-P Performance

The model of investigating the ASC-ASD-P Performance subscale (using transformed scores) showed a significant main effect of Time (*F*(2, 95.63) = 0.44, *p* = .65). As per Table 3, the between-group effect size at T1 was small and at T3 it was medium (with the control group having higher performance anxiety).

### PAS Social Anxiety and PAS Total

A significant main effect of Time was also shown in models for the PAS Social Anxiety (*F*(2, 95.27) = 3.51, *p* = .03) subscales and the PAS Total score (*F*(2, 94.78) = 3.19, *p* = .046), with reductions in scores indicated across time on both measures.

### Negative, Harmful, or Adverse Events

No parents reported any negative or adverse events from the activities or intervention during the intervention or at any of the follow-up assessments.

## Discussion

This is one of the first studies to evaluate whether a parent-mediated intervention specifically tailored to address anxiety in preschool-aged autistic children is effective at preventing or reducing child anxiety, child IU, or parental internalising problems at short-term or longer-term follow-up. The key finding was that children whose parents participated in the CLK-CUES intervention had significantly lower scores on an autism-specific measure of child anxiety post-intervention compared to baseline, specifically for anxiety around uncertainty. These benefits were noted at both short (x̅ = 4.99 weeks from T1) and longer term (x̅ = 14.89 months from T1) follow-up. Analyses which account for regression to the mean show a treatment effect of -3.55 points on the ASC-ASD-P Total score from T1-T2 and − 4.08 from T1-T3 and − 1.51 points on the ASC-ASD-P Uncertainty subscale score from T1-T2 and − 3.13 from T1-T3. The second key finding was that these benefits were specific to reductions in the aspects of child anxiety focussed upon in the intervention, as there were no significant differences between the intervention and control group on measures of child IU or parental internalising problems. The second aim of the study was to determine whether there were any negative or harmful effects of the CLK-CUES. No parents from either group reported any significant negative effects (adverse events) of either the intervention or being part of the trial.

There are many different types of interventions for reducing anxiety in autistic youth. Linden et al.’s (2023) meta-analysis of benefits and harms of interventions for mental health in autistic people concludes that CBT family-based interventions which are exposure focussed, such as CLK, have a standardised mean difference (SMD) of 2.24, indicating a significant benefit on levels of anxiety for anxious autistic youth (aged 2–17 years). However, Linden et al. noted that almost all the evidence is of very low certainty due to small sample sizes, methodological implications, or potential conflicts of interest. This study addresses some of these previous limitations. It was carefully designed so that the research team members conducting the assessments were not informed of the condition the child was in, and although the sample acquired in this study was below that initially set by the power analysis (*n* = 64) it remains relatively strong, being larger than 82% of the 406 clinical trials reported in Provenzani et al.’s (2020) systematic review of trials of interventions for autistic individuals. The sample is also larger than 84% of sample sizes in randomised controlled trials of CBT for autistic youth (Sharma et al., 2021). Additionally, the statistical approach taken (LMM) reduces issues of statistical power related to small sample sizes (Baayen et al., 2008). Despite the potential for being underpowered, the trial identified benefits for children of parents participating in the intervention group, with significant reductions in ASC-ASD-P Total and ASC-ASD-P Uncertainty subscale scores. This, combined with medium (ASC-ASD-P Total score) and large (Uncertainty subscale scores) effect sizes, indicates that the CLK-CUES intervention is a promising way to reduce and prevent anxiety, particularly anxiety measures on an autism-specific measure and anxiety around uncertainty, in autistic preschoolers. As children were recruited from the community (rather than from clinics) and thus had a wide range of levels of anxiety symptomology, the CLK-CUES shows potential to benefit a wide range of autistic preschoolers, not just those with significant or impairing levels of anxiety. Although statistical analyses were undertaken to evaluate the treatment effect whilst taking regression to the mean into account, the effect of CLK-CUES needs to be further evaluated in a fully powered trial, where anxiety levels are stratified during randomisation.

One interesting finding warranting discussion is the significant reduction on the ASC-ASD-P Uncertainty subscale for the intervention but not the control group post-intervention; however, no significant change on scores on the measures of IU (RULES, IUS-P). This may be because the ASC-ASD-P subscale focuses more on the worry around uncertainty whereas measures of IU focus more on the cognitive, emotional, and behavioural indicators of IU. As IU is an internal experience (Ong et al., 2023), parents may find it easier to provide ratings around the worry related to uncertainty than the IU itself. This may suggest that the preference for certainty, which is strongly associated with autism characteristics, remains but the anxiety around uncertainty reduces post-intervention or that the link between IU and anxiety may not be as strong in younger autistic children. Alternatively, it may be that incorporating some but not all elements of CUES into CLK-CUES was not sufficient to begin to address the cognitive elements of IU in autistic preschoolers. This explanation can be partly evaluated by comparing the change in scores on the IUS-P for preschoolers within CLK-CUES to the change in scores reported in Rodgers et al.’s (2023) feasibility outcomes of the CUES for school-aged autistic children aged 6–16 years who were receiving support for their anxiety through clinical services. The mean average change for the intervention group on the IUS-P in this study of CLK-CUES was − 2.2, which was almost identical to the change of -2.1 for the intervention in the Rodgers et al. trial of standard CUES. Therefore, the level of CUES content incorporated into CLK-CUES may be sufficient for supporting parents to reduce anxiety around uncertainty but there may need to be further content, possibly delivered as the children get older, enabling parents to support their children with the cognitive reactions to IU. Later “top up” sessions to build upon what parents learned when supporting their preschoolers may be beneficial and should be explored in future research.

Scores on the Performance Anxiety subscale of the ASC-ASD-P significantly increased over time, after the children started attending primary school. The Performance Anxiety items on the ASC-ASD-P include items with a social evaluation component, as well as items about avoiding mistakes or failure. Parents of school-aged children have previously described schoolwork and performance in school tasks as common triggers of anxiety (Simpson et al., 2020), with expectations changing as they settle into school and grow older (Ozsivadjian et al., 2012). The wide variability in methodology and measures used, the reliance on cross-sectional designs, and the lack of large enough samples to explore anxiety symptomatology by narrow age bands (see discussion in Varela et al., 2020) indicate a significant need for longitudinal, observational studies to document the trajectory of anxiety symptomatology for preschool into school-aged autistic children. This will help with planning autism-specific, age-relevant interventions as well as interpreting and evaluating their effectiveness.

The CLK-CUES intervention addresses a community priority for autism-specific interventions to reduce mental health difficulties. The incorporation of information around autism and autism-specific factors that increase the likelihood of anxiety, and distinguishing autism characteristics from anxiety, addresses the suggestions made by parents who received the standard CLK program in Bischoff et al. (2018). Additionally, the clinician-researcher running the groups had 15 years’ experience of working with autistic individuals experiencing mental health challenges. Systematic reviews have shown that the therapist’s level of experience in supporting autistic people with mental health challenges can be an enabler for or barrier to autistic people or their families accessing mental health services (Adams & Young, 2020; Cleary et al., 2022). When evaluating an autism-specific intervention, it is important to include outcome measures that are relevant and tailored to autistic people. This is supported by the findings of this study: the group who received the autism-specific intervention showed a significant reduction in anxiety scores on the ASC-ASD-P but not on the PAS. This supports previous suggestions that anxiety measures developed for and normed on neurotypical children are not calibrated to sensitively capture anxiety presentations in autistic people, and that those measures may result in underrepresentation or an insensitive measure of change in anxiety in young autistic children (Klein et al., 2023). As there are multiple autism-specific measures now available for anxiety (e.g., Rodgers et al., 2016, 2020; Scahill et al., 2019), as well as for other mental health challenges (e.g., Cassidy et al., 2021; Hedley et al., 2023), trials of mental health interventions for autistic people can and should include measures designed for autistic people. This will likely increase the relevance and trustworthiness of the research to the people who will benefit from it the most: autistic people and those who support them.

### Limitations and Future Directions

No study is without its limitations, and the findings of this study, whilst positive, should be considered in relation to its limitations. This study reports on the post-intervention outcomes which were, on average, collected 4.99 (short-term) and 14.89 months (longer term) post-baseline. Whilst the descriptor “longer term” is used to describe the second follow-up point, it is important to stress that the duration of this longer-term follow-up is front of mind when considering the conceptual and clinical implications of this work. Further studies following up these participants are needed to evaluate whether the benefits continue throughout childhood when anxiety rates in autistic youth typically increase, and the risk of multiple mental health conditions increases (Lai et al., 2019).

For research to be generalisable, the participants need to reflect the population from whom they are drawn. Therefore, it is important to reflect on the finding that families from the lower income brackets were overrepresented in those who did not attend their T2 and/or T3 assessment. It is important that researchers are aware of this risk, and potentially proactively incorporate strategies to recruit and retain participants who may find it difficult to attend. Community input may be needed to identify those strategies, as previous studies designed to recruit and implement intervention research in under-resourced populations report project attrition rates of over 60% (Carr & Lord, 2016). Although families within this project were provided with gift vouchers to thank them for their time, more flexible approaches to assessment and data collection which match family schedules and needs may also be needed.

Whilst participants were randomly allocated to the intervention and control groups, the level of anxiety in the intervention group was significantly higher than in the control group at T1. Therefore, it may be that the intervention group showed regression to the mean. However, there are multiple aspects that suggest that the intervention had a significant effect. These include (1) the significant decline in ASC-ASD Total and Uncertainty scores was immediately post-intervention (i.e., not a regression over T2 and T3 towards the mean) (2) analysis which take into account regression to the mean indicate at treatment effect of 3–4 point reduction on the ASC-ASD-P (3) the between group effect sizes for ASC-ASD Performance Anxiety scores show a medium effect size, indicating a benefit to the intervention group, after the children started school, and (4) the between group effect sizes for the PAS Total score in Table 3 do not significantly differ between groups at T1 but show a large effect size at T2 (and medium effect size at T3), with benefits for anxiety in the intervention group. Combined these all suggest that there is promising evidence that the intervention had a positive effect on reducing uncertainty and potentially preventing performance anxiety in young autistic children.

The study did not manage to recruit the 64 participants suggested by the a priori power analysis. The narrow child age range (4–5 years), the need for parents to attend groups face-to-face, and recruiting during a global pandemic may have impacted recruitment. Whilst the aim was for a fully powered randomised controlled trial, the sample size of 59 means this study could be considered a pilot study. Future studies could recruit larger samples for a fully powered trial through multi-site collaboration or through delivery of the intervention via telehealth, both of which would extend the geographical range from which participants could participate. The standard CLK has been shown to be effective at reducing anxiety in inhibited neurotypical children when delivered online (Morgan et al., 2016, 2017). However, as Morgan et al. (2016) showed that less than 40% of parents completed all modules, it may be that weekly online parent groups could be beneficial at supporting parents through the process. This would also then allow the group to still have the benefit of parent peer-to-peer support, identified by parents as important in the pilot testing of CLK-CUES (Simpson et al., 2023).

Although the study did not place any exclusions on participants based upon co-occurring conditions, there was a limited number of child participants who were reported to have a co-occurring ID (3.5% formally diagnosed, 3.5% mentioned/informally diagnosed). Anxiety in autistic individuals with co-occurring ID is challenging to diagnose and often overlooked (Appleton et al., 2019; Winder-Patel et al., 2022). Diagnostic overshadowing and a lack of research detailing the profile of anxiety symptomatology specifically in autistic individuals with co-occurring ID has led to a lack of specific measures that focus on anxiety in autistic individuals with ID. Such measures will allow for a nuanced and thorough understanding of anxiety so that the trajectory of anxiety symptomatology in this population can be examined over time (Edwards et al., 2022). Because anxiety may not be fully understood in autistic children with co-occurring ID, and because of an ongoing history of being excluded from autism research studies (Russell et al., 2019), it may be that parents of this population automatically assumed that this study would not be relevant or beneficial for them. Studies that wish to include autistic participants with co-occurring ID, or any complex support or communication need, could make the opportunity for these groups to participate very clear on recruitment materials. It is important for researchers to proactively collaborate early on with autistic and autism community groups that include individuals with ID or complex support/communication needs to ensure their study design is advised by and supportive of this group.

As this trial was designed to evaluate the potential for this intervention to benefit young autistic children with a wide variety of levels of anxiety symptomatology, the decision was taken to use both an autism-specific anxiety questionnaire and an anxiety questionnaire designed for neurotypical children. Future studies may wish to select a single primary outcome measure rather than having multiple primaries. Alternatively, they may wish to use a diagnostic tool such as Anxiety Disorders Interview Schedule—Autism Spectrum Addendum (ADIS-ASA; see Kerns et al., 2017) to evaluate the efficacy of the intervention in preventing anxiety disorders as well as in reducing anxiety symptomatology. As significant or impairing anxiety was not a criterion for participation, some participants had relatively low scores on the measures of anxiety, making the overall intervention group reduction in anxiety scores even more notable. Although the decision was taken to focus upon parent-report rather than child self-report to enable children of all ability levels to participate, future studies may wish to incorporate both parent- and child-report where possible. Finally, the study only reports upon anxiety measures which use parent-report, which has limitations as the informants were aware if they were in the intervention or control group. Whilst parent-report questionnaires are useful, future studies may also wish to evaluate intervention outcomes through other informants or parent and child interviews which can document the lived experience. For parents, this could also include an assessment of fidelity or real-world gains, for example, in knowledge of the strategies used.

## Conclusion

This pilot RCT indicates promise for CLK-CUES; an autism-specific, parent-mediated program designed to reduce or prevent anxiety in autistic preschoolers. However, fully powered trials with larger samples are required to fully evaluate the effects. These trials should use autism-specific measures of anxiety, and where possible collect data from parents and children, potentially through formal diagnostic processes (e.g. ADIS) as well as questionnaires. Further collaboration with the autistic and autism community will also allow for further improvements to CLK-CUES and to the design for the fully powered RCT.

## Electronic supplementary material

Below is the link to the electronic supplementary material.

Supplementary Material 1

Supplementary Material 2

## References

[CR1] Adams, D., Clark, M., & Keen, D. (2019a). Using self-report to explore the relationship between anxiety and quality of life in children on the autism spectrum. *Autism Research*, *12*(10), 1505–1515. 10.1002/aur.2155 31207183 10.1002/aur.2155

[CR65] Adams, D., Malone, S., Simpson, K., Tucker, M., Rapee, R. M., Rodgers, J., & Keen, D. (2021). Protocol for a longitudinal study investigating the role of anxiety on academic outcomes in children on the autism spectrum. *PLoS ONE*, *16*(9), e0257223. 10.1371/journal.pone.0257223 10.1371/journal.pone.0257223 PMC8445440 34529686

[CR2] Adams, D., Simpson, K., & Keen, D. (2020). Exploring anxiety at home, school, and in the community through self-report from children on the autism spectrum. *Autism Research*, *13*(4), 603–614. 10.1002/aur.2246 31793245 10.1002/aur.2246

[CR3] Adams, D., & Young, K. (2020). A systematic review of the perceived barriers and facilitators to accessing psychological treatment for mental health problems in individuals on the autism spectrum. *Review Journal of Autism and Developmental Disorders*, 1–18. 10.1007/s40489-020-00226-7

[CR4] Adams, D., Young, K., Simpson, K., & Keen, D. (2019b). Parent descriptions of the presentation and management of anxiousness in children on the autism spectrum. *Autism*, *23*(4), 980–992. 10.1177/1362361318794031 30114931 10.1177/1362361318794031

[CR5] Ambrose, K., Simpson, K., & Adams, D. (2021). The impact of anxiety on the participation of children on the autism spectrum. *Journal of Autism and Developmental Disorders*, *52*(7), 2958–2969. 10.1007/s10803-021-05162-x 34196892 10.1007/s10803-021-05162-x

[CR6] Appleton, H., Roberts, J., & Simpson, K. (2019). How is anxiety identified and diagnosed in individuals with autism spectrum disorder and intellectual disability? A scoping review. *Journal of Mental Health Research in Intellectual Disabilities*, *12*(3–4), 152–175. 10.1080/19315864.2019.1679299

[CR7] Baayen, R., Davidson, D., & Bates, D. (2008). Mixed-effects modeling with crossed random effects for subjects and items. *Journal of Memory and Language*, *59*(4), 390–412. 10.1016/j.jml.2007.12.005

[CR8] Baribeau, D. A., Vigod, S., Pullenayegum, E., Kerns, C. M., Mirenda, P., Smith, I. M., Vaillancourt, T., Volden, J., Waddell, C., Zwaigenbaum, L., Bennett, T., Duku, E., Elsabbagh, M., Georgiades, S., Ungar, W. J., Zaidman-Zait, A., & Szatmari, P. (2020). Repetitive behavior severity as an early indicator of risk for elevated anxiety symptoms in autism spectrum disorder. *Journal of the American Academy of Child & Adolescent Psychiatry*, *59*(7), 890–899. 10.1016/j.jaac.2019.08.478 31541676 10.1016/j.jaac.2019.08.478

[CR9] Baribeau, D. A., Vigod, S., Pullenayegum, E., Kerns, C. M., Mirenda, P., Smith, I. M., Vaillancourt, T., Volden, J., Waddell, C., Zwaigenbaum, L., Bennett, T., Duku, E., Elsabbagh, M., Georgiades, S., Ungar, W. J., Zaidman-Zait, A., & Szatmari, P. (2021). Co-occurring trajectories of anxiety and insistence on sameness behaviour in autism spectrum disorder. *The British Journal of Psychiatry*, *218*(1), 20–27. 10.1192/bjp.2020.127 32641181 10.1192/bjp.2020.127

[CR10] Baribeau, D. A., Vigod, S. N., Pullenayegum, E., Kerns, C. M., Vaillancourt, T., Duku, E., Smith, I. M., Volden, J., Zwaigenbaum, L., Bennett, T., Elsabbagh, M., Zaidman-Zait, A., Richard, A. E., & Szatmari, P. (2022). Developmental cascades between insistence on sameness behaviour and anxiety symptoms in autism spectrum disorder. *European Child & Adolescent Psychiatry*. 10.1007/s00787-022-02049-9. ePub ahead of print. 10.1007/s00787-022-02049-9 35871413

[CR11] Barnett, A. G., Van Der Pols, J. C., & Dobson, A. J. (2004). Regression to the mean: What it is and how to deal with it. *International Journal of Epidemiology*, *34*(1), 215–220. 10.1093/ije/dyh299 15333621 10.1093/ije/dyh299

[CR12] Bird, S., Moid, L. E., Jones, C. A., & Surtees, A. D. (2024). The relationships between restrictive/repetitive behaviours, intolerance of uncertainty, and anxiety in autism: A systematic review and meta-analysis. *Research in Autism Spectrum Disorders*, *117*, 102428. 10.1016/j.rasd.2024.102428

[CR13] Bischof, N. L., Rapee, R. M., Hudry, K., & Bayer, J. K. (2018). Acceptability and caregiver-reported outcomes for young children with autism spectrum disorder whose parents attended a preventative population‐based intervention for anxiety: A pilot study. *Autism Research*, *11*(8), 1166–1174. 10.1002/aur.1963 29761836 10.1002/aur.1963

[CR14] Boulter, C., Freeston, M., South, M., & Rodgers, J. (2014). Intolerance of uncertainty as a framework for understanding anxiety in children and adolescents with autism spectrum disorders. *Journal of Autism and Developmental Disorders*, *44*(6), 1391–1402. 10.1007/s10803-013-2001-x 24272526 10.1007/s10803-013-2001-x

[CR15] Brede, J., Cage, E., Trott, J., Palmer, L., Smith, A., Serpell, L., Mandy, W., & Russell, A. (2022). We have to try to find a way, a clinical bridge-autistic adults’ experience of accessing and receiving support for mental health difficulties: A systematic review and thematic meta-synthesis. *Clinical Psychology Review*, *93*, 102131. 10.1016/j.cpr.2022.102131 35180632 10.1016/j.cpr.2022.102131

[CR16] Carr, T., & Lord, C. (2016). A pilot study promoting participation of families with limited resources in early autism intervention. *Research in Autism Spectrum Disorders*, *25*, 87–96. 10.1016/j.rasd.2016.02.003 10.1016/j.rasd.2016.02.003 PMC4804337 27019670

[CR17] Cassidy, S. A., Bradley, L., Cogger-Ward, H., & Rodgers, J. (2021). Development and validation of the suicidal behaviours questionnaire-autism spectrum conditions in a community sample of autistic, possibly autistic and non-autistic adults. *Molecular Autism*, *12*(1), 46. 10.1186/s13229-021-00449-3 34154642 10.1186/s13229-021-00449-3 PMC8218414

[CR18] Chan, N., Sanner, C. M., McGregor, H. A., Preston, A. E., & Neece, C. L. (2021). Anxiety in a preschool-aged sample with autism spectrum disorder and developmental delay: Rates, symptom manifestation, and parenting risk variables. *Journal of Mental Health Research in Intellectual Disabilities*, *14*(2), 202–224. 10.1080/19315864.2021.1883781

[CR19] Cleary, M., West, S., Hunt, G. E., McLean, L., Hungerford, C., & Kornhaber, R. (2022). How people with autism access mental health services specifically suicide hotlines and crisis support services, and current approaches to mental health care: A scoping review. *Issues in Mental Health Nursing*, *43*(12), 1093–1106. 10.1080/01612840.2022.2108529 36041121 10.1080/01612840.2022.2108529

[CR20] Cohen, J. (1988). *Statistical power analysis for the behavioral sciences* (2nd ed.). L. Erlbaum Associates.

[CR21] den Houting, J., Adams, D., Roberts, J., & Keen, D. (2019). An exploration of autism-specific and non‐autism‐specific measures of anxiety symptomatology in school‐aged autistic children. *Clinical Psychologist*, *23*(3), 237–248. 10.1111/cp.12174

[CR22] Doyle, F. L., Dodd, H. F., Morris, T. M., Lazarus, R. S., Byrow, Y., & Hudson, J. L. (2021). Targeting risk factors for inhibited preschool children: An anxiety prevention program. *Behaviour Research and Therapy*, *147*, 103982. 10.1016/j.brat.2021.103982 34678709 10.1016/j.brat.2021.103982

[CR23] Driscoll, K., Schonberg, M., Stark, M. F., Carter, A. S., & Hirshfeld-Becker, D. (2020). Family-centered cognitive behavioral therapy for anxiety in very young children with autism spectrum disorder. *Journal of Autism and Developmental Disorders*, *50*, 3905–3920. 10.1007/s10803-020-04446-y 32146598 10.1007/s10803-020-04446-y

[CR25] Edwards, S. L., Rapee, R. M., Kennedy, S. J., & Spence, S. H. (2010). The assessment of anxiety symptoms in preschool-aged children: The revised preschool anxiety scale. *Journal of Clinical Child & Adolescent Psychology*, *39¸*, 400–409. 10.1080/15374411003691701 10.1080/15374411003691701 20419580

[CR24] Edwards, G., Tarver, J., Shelley, L., Bird, M., Hughes, J., Crawford, H., & Waite, J. (2022). Utilising interview methodology to inform the development of new clinical assessment tools for anxiety in autistic individuals who speak few or no words. *Journal of Autism and Developmental Disorders*, *epub ahead of print*. 10.1007/s10803-022-05509-y 10.1007/s10803-022-05509-y PMC10229722 35304663

[CR26] Hedley, D., Batterham, P. J., Bury, S. M., Clapperton, A., Denney, K., Dissanayake, C., Fox, P., Frazier, T. W., Gallagher, E., Hayward, S. M., Robinson, J., Sahin, E., Trollor, J., Uljarevic, M., & Stokes, M. A. (2023). The suicidal ideation attributes scale-modified (SIDAS-M): Development and preliminary validation of a new scale for the measurement of suicidal ideation in autistic adults. *Autism*, *27*(4), 1115–1131. 10.1177/13623613221131234 36237153 10.1177/13623613221131234

[CR27] Jenkinson, R., Milne, E., & Thompson, A. (2020). The relationship between intolerance of uncertainty and anxiety in autism: A systematic literature review and meta-analysis. *Autism*, *24*(8), 1933–1944. 10.1177/1362361320932437 32564625 10.1177/1362361320932437 PMC7539603

[CR28] Keefer, A., Kreiser, N. L., Singh, V., Blakeley-Smith, A., Duncan, A., Johnson, C., Klinger, L., Meyer, A., Reaven, J., & Vasa, R. A. (2016). Intolerance of uncertainty predicts anxiety outcomes following CBT in Youth with ASD. *Journal of Autism and Developmental Disorders*, *47*(12), 3949–3958. 10.1007/s10803-016-2852-z 10.1007/s10803-016-2852-z 27405445

[CR29] Keen, D., Adams, D., Simpson, K., den Houting, J., & Roberts, J. (2019). Anxiety-related symptomatology in young children on the autism spectrum. *Autism*, *23*(2), 350–358. 10.1177/1362361317734692 29202607 10.1177/1362361317734692

[CR30] Kerns, C. M., Kendall, P. C., Zickgraf, H., Franklin, M. E., Miller, J., & Herrington, J. (2015). Not to be overshadowed or overlooked: Functional impairments associated with comorbid anxiety disorders in youth with ASD. *Behavior Therapy*, *46*(1), 29–39. 10.1016/j.beth.2014.03.005 25526833 10.1016/j.beth.2014.03.005

[CR31] Kerns, C. M., Renno, P., Kendall, P. C., Wood, J. J., & Storch, E. A. (2017). Anxiety disorders interview schedule–autism addendum: Reliability and validity in children with autism spectrum disorder. *Journal of Clinical Child & Adolescent Psychology*, *46*(1), 88–100. 10.1080/15374416.2016.1233501 27925775 10.1080/15374416.2016.1233501 PMC5441235

[CR32] Klein, J., Kerns, C., Hills, K., Hogan, A., Matherly, S., & Roberts, J. (2023). Brief report: Prevalence and predictors of DSM-specific and distinct anxiety in cognitively impaired autistic preschool children. *Journal of Autism and Developmental Disorders*, 1–9. 10.1007/s10803-023-05978-9 10.1007/s10803-023-05978-9 PMC11441628 37039980

[CR33] Lai, M. C., Kassee, C., Besney, R., Bonato, S., Hull, L., Mandy, W., Szatmari, P., & Ameis, S. H. (2019). Prevalence of co-occurring mental health diagnoses in the autism population: A systematic review and meta-analysis. *The Lancet Psychiatry*, *6*(10), 819–829. 10.1016/S2215-0366(19)30289-5 31447415 10.1016/S2215-0366(19)30289-5

[CR34] Lin, L. Y., & Huang, P. C. (2019). Quality of life and its related factors for adults with autism spectrum disorder. *Disability and Rehabilitation*, *41*(8), 896–903. 10.1080/09638288.2017.1414887 29228834 10.1080/09638288.2017.1414887

[CR35] Linden, A., Best, L., Elise, F., Roberts, D., Branagan, A., Tay, Y. B. E., Crane, L., Cusack, J., Davidson, B., Hearst, C., Mandy, W., Rai, D., Smith, E., & Gurusamy, K. (2023). Benefits and harms of interventions to improve anxiety, depression, and other mental health outcomes for autistic people: A systematic review and network meta-analysis of randomised controlled trials. *Autism*, *27*(1), 7–30. 10.1177/13623613221117931 35957523 10.1177/13623613221117931 PMC9806485

[CR36] Lovibond, S. H., & Lovibond, P. F. (1995). *Depression anxiety stress scales (DASS–21, DASS–42)*. APA PsycTests. [Database record].

[CR37] Mandy, W. (2022). Six ideas about how to address the autism mental health crisis. *Autism: The International Journal of Research and Practice*, *26*(2), 289–292. 35109701 10.1177/13623613211067928

[CR38] Morgan, A. J., Rapee, R. M., & Bayer, J. K. (2016). Prevention and early intervention of anxiety problems in young children: A pilot evaluation of cool little kids online. *Internet Interventions*, *4*, 105–112. 10.1016/j.invent.2016.05.001 30135796 10.1016/j.invent.2016.05.001 PMC6096126

[CR39] Morgan, A. J., Rapee, R. M., Salim, A., Goharpey, N., Tamir, E., McLellan, L. F., & Bayer, J. K. (2017). Internet-delivered parenting program for prevention and early intervention of anxiety problems in young children: Randomized controlled trial. *Journal of the American Academy of Child & Adolescent Psychiatry*, *56*(5), 417–425. 10.1016/j.jaac.2017.02.010 28433091 10.1016/j.jaac.2017.02.010

[CR40] Neil, L., Olsson, N. C., & Pellicano, E. (2016). The relationship between intolerance of uncertainty, sensory sensitivities, and anxiety in autistic and typically developing children. *Journal of Autism and Developmental Disorders*, *46*, 1962–1973. 10.1007/s10803-016-2721-9 26864157 10.1007/s10803-016-2721-9 PMC4860201

[CR41] Ong, C. S., Magiati, I., Maybery, M. T., Rodgers, J., Uljarevic, M., & Alvares, G. A. (2023). Parental perspectives of the everyday experiences of uncertainty among young children on the autism spectrum. *Research in Autism Spectrum Disorders*, *101*, 102087. 10.1016/j.rasd.2022.102087

[CR42] Ooi, J., Dodd, H. F., Meiser-Stedman, R., Hudson, J. L., Bridges, J., & Pass, L. (2022). The efficacy of interventions for behaviourally inhibited preschool-aged children: A meta-analysis. *Journal of Anxiety Disorders*, *88*, 102559. 10.1016/j.janxdis.2022.102559 35366584 10.1016/j.janxdis.2022.102559

[CR43] Ozsivadjian, A., Knott, F., & Magiati, I. (2012). Parent and child perspectives on the nature of anxiety in children and young people with autism spectrum disorders: A focus group study. *Autism*, *16*(2), 107–121. 10.1177/1362361311431703 22297200 10.1177/1362361311431703

[CR44] Provenzani, U., Fusar-Poli, L., Brondino, N., Damiani, S., Vercesi, M., Meyer, N., Rocchetti, M., & Politi, P. (2020). What are we targeting when we treat autism spectrum disorder? A systematic review of 406 clinical trials. *Autism*, *24*(2), 274–284. 10.1177/1362361319854641 31269800 10.1177/1362361319854641

[CR45] Rapee, R. M. (2013). The preventative effects of a brief, early intervention for preschool-aged children at risk for internalising: Follow‐up into middle adolescence. *Journal of Child Psychology and Psychiatry*, *54*(7), 780–788. 10.1111/jcpp.12048 23397984 10.1111/jcpp.12048

[CR49] Rodgers, J., Wigham, S., McConachie, H., Freeston, M., Honey, E., & Parr, J. R. (2016). Development of the anxiety scale for children with Autism Spectrum disorder (ASC-ASD). *Autism Research*, *9*, 1205–1215. 10.1002/aur.1603 26887910 10.1002/aur.1603

[CR48] Rodgers, J., Goodwin, J., Parr, J. R., Grahame, V., Wright, C., Padget, J., Garland, D., Osborne, M., Labus, M., Kernohan, A., & Freeston, M. (2019). Coping with uncertainty in everyday situations (CUES©) to address intolerance of uncertainty in autistic children: Study protocol for an intervention feasibility trial. *Trials*, *20*(1), 1–11. 10.1186/s13063-019-3479-0 31248435 10.1186/s13063-019-3479-0 PMC6598241

[CR46] Rodgers, J., Farquhar, K., Mason, D., Brice, S., Wigham, S., Ingham, B., Freeston, M., & Parr, J. R. (2020). Development and initial evaluation of the anxiety scale for autism – adults. *Autism in Adulthood*, *2*(1), 24–33. 10.1089/aut.2019.0044 36600985 10.1089/aut.2019.0044 PMC8992845

[CR47] Rodgers, J., Goodwin, J., Garland, D., Grahame, V., Isard, L., Kernohan, A., Labus, M., Osborne, M., Parr, J. R., Rob, P., Wright, C., & Freeston, M. (2023). Coping with uncertainty in everyday situations (CUES©) to address intolerance of uncertainty in autistic children: An intervention feasibility trial. *Journal of Autism and Developmental Disorders*, *53*(9), 3460–3474. 10.1007/s10803-022-05645-5 35790596 10.1007/s10803-022-05645-5 PMC10465370

[CR50] Russell, G., Mandy, W., Elliott, D., White, R., Pittwood, T., & Ford, T. (2019). Selection bias on intellectual ability in autism research: A cross-sectional review and meta-analysis. *Molecular Autism*, *10*(1), 1–10. 10.1186/s13229-019-0260-x 30867896 10.1186/s13229-019-0260-x PMC6397505

[CR51] Sanchez, A. L., Cornacchio, D., Chou, T., Leyfer, O., Coxe, S., Pincus, D., & Comer, J. S. (2017). Development of a scale to evaluate young children’s responses to uncertainty and low environmental structure. *Journal of Anxiety Disorders*, *45*, 17–23. 10.1016/j.janxdis.2016.11.006 27907833 10.1016/j.janxdis.2016.11.006

[CR52] Scahill, L., Lecavalier, L., Schultz, R. T., Evans, A. N., Maddox, B., Pritchett, J., Herringon, J., Gillespie, S., Miller, J., Amoss, T., Aman, M. G., Bearss, K., Gadow, K., & Edwards, M. C. (2019). Development of the parent-rated anxiety scale for youth with autism spectrum disorder. *Journal of the American Academy of Child & Adolescent Psychiatry*, *58*(9), 887–896. 10.1016/j.jaac.2018.10.016 30797036 10.1016/j.jaac.2018.10.016

[CR53] Sharma, S., Hucker, A., Matthews, T., Grohmann, D., & Laws, K. R. (2021). Cognitive behavioural therapy for anxiety in children and young people on the autism spectrum: A systematic review and meta-analysis. *BMC Psychology*, *9*(1), 1–16. 10.1186/s40359-021-00658-8 34598734 10.1186/s40359-021-00658-8 PMC8487131

[CR66] Simpson, K., Adams, D., Malone, S., Tucker, M., Rapee, R. M., & Rodgers, J. (2023). A parent-mediated anxiety intervention specifically tailored for autistic preschoolers: A pilot study. *The American Journal of Occupational Therapy*, *77*(2), 7702185100 10.5014/ajot.2023.050031 37018051

[CR54] Simpson, K., Adams, D., Wheeley, E., & Keen, D. (2020). Parent perspectives on the presentation, triggers, impact, and support of anxiety in young children on the autism spectrum. *Journal of Child and Family Studies*, *29*, 572–582. 10.1007/s10826-019-01576-5

[CR55] Townsend, A. N., Guzick, A. G., Hertz, A. G., Kerns, C. M., Goodman, W. K., Berry, L. N., Kendall, P. C., Wood, J. J., & Storch, E. A. (2022). Anger outbursts in youth with ASD and anxiety: Phenomenology and relationship with family accommodation. *Child Psychiatry & Human Development*, 1–10. 10.1007/s10578-022-01489-3 10.1007/s10578-022-01489-3 PMC10300226 36576640

[CR56] Ung, D., Selles, R., Small, B. J., & Storch, E. A. (2015). A systematic review and meta-analysis of cognitive-behavioral therapy for anxiety in youth with high-functioning autism spectrum disorders. *Child Psychiatry & Human Development*, *46*, 533–547. 10.1007/s10578-014-0494-y 25246292 10.1007/s10578-014-0494-y

[CR57] van Steensel, F. J., Bogels, S. M., & Perrin, S. (2011). Anxiety disorders in children and adolescents with autistic spectrum disorders: A meta-analysis. *Clinical Child and Family Psychology Review*, *14*, 302–317. 10.1007/s10567-011-0097-0 21735077 10.1007/s10567-011-0097-0 PMC3162631

[CR58] Varela, R. E., DuPont, R., Kamps, J. L., Weems, C. F., Niditch, L., Beaton, E. A., & Pucci, G. (2020). Age differences in expression of generalized and social anxiety among youth with autism spectrum disorder. *Journal of Autism and Developmental Disorders*, *50*, 730–740. 10.1007/s10803-019-04289-2 31729598 10.1007/s10803-019-04289-2

[CR59] Vasa, R. A., Keefer, A., McDonald, R. G., Hunsche, M. C., & Kerns, C. M. (2020). A scoping review of anxiety in young children with autism spectrum disorder. *Autism Research*, *13*(12), 2038–2057. 10.1002/aur.2395 32978905 10.1002/aur.2395

[CR60] Vasa, R. A., Kerns, C. M., Singh, V., McDonald, R., Jang, Y. S., & Keefer, A. (2023). Anxiety in autistic preschool children: Phenomenology and a network analysis of correlates. *Autism Research*, *6*, 1561–1572. 10.1002/aur.2968 10.1002/aur.2968 37350221

[CR62] Walker, S. (2009). *What do we know about the relationship between intolerance of uncertainty and worry in young children?* (Unpublished Thesis, Newastle University, Newcastle Upon Tyne).

[CR61] Walker, E. A., Redfern, A., & Oleson, J. J. (2019). Linear mixed-model analysis to examine longitudinal trajectories in vocabulary depth and breadth in children who are hard of hearing. *Journal of Speech Language and Hearing Research*, *62*(3), 525–542. 10.1044/2018_JSLHR-L-ASTM-18-0250 10.1044/2018_JSLHR-L-ASTM-18-0250 PMC6802902 30950738

[CR63] Winder-Patel, B., Tudor, M. E., Kerns, C. M., Davis, K., Nordahl, C. W., Amaral, D. G., & Solomon, M. (2022). Often undiagnosed but treatable: Case vignettes and clinical considerations for assessing anxiety disorders in youth with autism spectrum disorder and intellectual disability. *Evidence-Based Practice in Child and Adolescent Mental Health*, *7*(1), 24–40. 10.1080/23794925.2021.1923090 35284637 10.1080/23794925.2021.1923090 PMC8916744

[CR64] Wood, J. J., Kendall, P. C., Wood, K. S., Kerns, C. M., Seltzer, M., Small, B. J., Lewin, A. B., & Storch, E. A. (2020). Cognitive behavioral treatments for anxiety in children with autism spectrum disorder. *JAMA Psychiatry*, *77*(5), 474. 10.1001/jamapsychiatry.2019.4160 31755906 10.1001/jamapsychiatry.2019.4160 PMC6902190

