# Supplementary material for: Prevention and Reduction of Anxiety in Autistic Preschoolers Through an Autism-Specific Parent-Mediated Intervention: A Pilot Randomised Controlled Trial Evaluating Short and Longer Term Outcomes
Source: J Autism Dev Disord. 2024 Sep 26;56(2):447–63. doi: 10.1007/s10803-024-06570-5 (PMC12864335; doi:10.1007/s10803-024-06570-5)
Supplement: Supplementary file 1 — Supplementary Material 1 [file 10803_2024_6570_MOESM1_ESM.docx]

**Supplementary Table 1.** Mean (*SD*) of secondary outcome variables for the control and intervention group at each time point and size of between-group effects, alongside results of linear Mixed Models Mean

| Variable |  | Time 1 | | | Time 2 | | | Time 3 | | | Results of Linear Mixed Model (LMM) | | |
| --- | --- | --- | --- | --- | --- | --- | --- | --- | --- | --- | --- | --- | --- |
|  |  | Control  *M(SD)* | Intervention  *M(SD)* | Between-group effect size | Control  *M(SD)* | Intervention  *M(SD)* | Between-group effect size | Control  *M(SD)* | Intervention  *M(SD)* | Between-group effect size | Time | Group | Group*Time |
| IU | RULES^1^ | 53.36  (16.88) | 55.48  (14.38) | .39 | 53.42 (16.37) | 53.48 (15.79) | -.01 | 50.25 (17.23) | 53.55 (13.55) | .64 | *F*(2, 94.25) = .95, *p* = .39 | *F*(1, 55.92) = .58, *p* = .45 | *F*(2, 94.25) = 1.22, *p* = .30 |
|  | IUS-P | 35.89  (13.31) | 37.96  (12.06) | -.33 | 33.79 (11.96) | 35.77 (11.38) | -.42 | 33.25 (12.69) | 36.91 (10.96) | -.69 | *F*(2, 95.29) = 2.09, *p* = .13 | *F*(1, 54.44) = 1.08, *p* = .30 | *F*(2, 95.29) = .43, *p* = .65 |
| DASS | Depression | 9.55 (9.67) | 10.20  (7.80) | -.13 | 8.91 (9.08) | 8.96  (8.06) | .13 | 11.31 (9.36) | 10.78 (10.34) | -.07 | *F*(2, 92.30) = .46, *p* = .63 | *F*(1, 52.42) = .004, *p* = .95 | *F*(2, 92.30) = .23, *p* = .79 |
|  | Anxiety | 6.67 (6.11) | 7.38  (7.35) | -.14 | 8.50 (7.96) | 8.00  (8.64) | .13 | 8.75 (8.08) | 7.21  (9.06) | .36 | *F*(2, 97.39) = .80, *p* = .45 | *F*(1, 57.10) = .12, *p* = .73 | *F*(2, 97.39) = .77, *p* = .47 |
|  | Stress | 17.03 (9.29) | 18.48  (8.01) | -.30 | 18.17 (9.72) | 17.44 (11.54) | .26 | 18.08 (11.53) | 17.65  (9.90) | .12 | *F*(2, 93.25) = .04, *p* = .96 | *F*(1, 54.82) = .003, *p* = .96 | *F*(2, 93.25) = 1.07, *p* = .35 |

^1^ Scores were transformed for analysis but raw scores presented here; effect sizes reflect transformed scores.
