# Supplementary material for: Prevention and Reduction of Anxiety in Autistic Preschoolers Through an Autism-Specific Parent-Mediated Intervention: A Pilot Randomised Controlled Trial Evaluating Short and Longer Term Outcomes
Source: J Autism Dev Disord. 2024 Sep 26;56(2):447–63. doi: 10.1007/s10803-024-06570-5 (PMC12864335; doi:10.1007/s10803-024-06570-5)
Supplement: Supplementary file 2 — Supplementary Material 2 [file 10803_2024_6570_MOESM2_ESM.docx]

Supplementary material Table 2-4: correlations between primary outcome measures

Table 2: Time 1 correlations between primary outcome measures

|  | | ASC-ASD-P | | | | |
| --- | --- | --- | --- | --- | --- | --- |
|  |  | Total | Uncertainty | Performance | Anxious Arousal | Separation Anxiety |
| ASC-ASD-P | Total score | - |  |  |  |  |
|  | Uncertainty | .866 | - |  |  |  |
|  | Performance anxiety | .792 | .558 | - |  |  |
|  | Anxious Arousal | .676 | .395 | .447 | - |  |
|  | Separation Anxiety | .802 | .595 | .468 | .547 | - |
| PAS-R | Total score | .837 | .71 | .687 | .503 | .726 |

Table 3: Time 2 correlations between primary outcome measures

|  | | ASC-ASD-P | | | | |
| --- | --- | --- | --- | --- | --- | --- |
|  |  | Total | Uncertainty | Performance | Anxious Arousal | Separation Anxiety |
| ASC-ASD-P | Total score | - |  |  |  |  |
|  | Uncertainty | .887 | - |  |  |  |
|  | Performance anxiety | .788 | .579 | - |  |  |
|  | Anxious Arousal | .736 | .584 | .496 | - |  |
|  | Separation Anxiety | .754 | .533 | .447 | .442 | - |
| PAS-R | Total score | .786 | .689 | .583 | .579 | .645 |

Table 4: Time 3 correlations between primary outcome measures

|  | | ASC-ASD-P | | | | |
| --- | --- | --- | --- | --- | --- | --- |
|  |  | Total | Uncertainty | Performance | Anxious Arousal | Separation Anxiety |
| ASC-ASD-P | Total score | - |  |  |  |  |
|  | Uncertainty | .795 | - |  |  |  |
|  | Performance anxiety | .758 | .535 | - |  |  |
|  | Anxious Arousal | .621 | .461 | .239 | - |  |
|  | Separation Anxiety | .713 | .469 | .284 | .495 | - |
| PAS-R | Total score | .863 | .666 | .669 | .594 | .693 |
